# Quantitative Localization Microscopy: Effects of Photophysics and Labeling Stoichiometry

**DOI:** 10.1371/journal.pone.0127989

**Published:** 2015-05-20

**Authors:** Robert P. J. Nieuwenhuizen, Mark Bates, Anna Szymborska, Keith A. Lidke, Bernd Rieger, Sjoerd Stallinga

**Affiliations:** 1 Quantitative Imaging Group, Delft University of Technology, Delft, The Netherlands; 2 Department of NanoBiophotonics, Max Planck Institute for Biophysical Chemistry, Göttingen, Germany; 3 Cell Biology and Biophysics Unit, European Molecular Biology Laboratory, Heidelberg, Germany; 4 Department of Physics and Astronomy, University of New Mexico, Albuquerque, New Mexico, USA; Julius-Maximilians-University Würzburg, GERMANY

## Abstract

Quantification in localization microscopy with reversibly switchable fluorophores is severely hampered by the unknown number of switching cycles a fluorophore undergoes and the unknown stoichiometry of fluorophores on a marker such as an antibody. We overcome this problem by measuring the average number of localizations per fluorophore, or generally per fluorescently labeled site from the build-up of spatial image correlation during acquisition. To this end we employ a model for the interplay between the statistics of activation, bleaching, and labeling stoichiometry. We validated our method using single fluorophore labeled DNA oligomers and multiple-labeled neutravidin tetramers where we find a counting error of less than 17% without any calibration of transition rates. Furthermore, we demonstrated our quantification method on nanobody- and antibody-labeled biological specimens.

## Introduction

Localization microscopy (e.g. PALM/STORM) is a powerful tool for imaging biological structures on the nanoscale [1–5]. In order to yield information about the molecular composition of the sample, localization microscopy images must be quantifiable in terms of the density of fluorescently labeled molecules or of binding sites. The relationship between these desired densities and the actual measured density of localizations is non-trivial however, since the (average) number of localizations per fluorophore and the labeling stoichiometry are unknown.

The use of photo-activatable fluorescent proteins (FPs) [1, 3, 6] offers a relatively direct approach to counting and thus to obtaining the desired densities, provided they switch off irreversibly after a non-interrupted on-state. In practice, however, there are several factors that can either lead to overcounting or undercounting of molecules [7]. Overcounting occurs when molecules are localized several times, either due to short-term blinking during the on-state or due to long lived dark states that effectively lead to reversible switching of FPs [8, 9]. In addition, overexpression of fluorescent fusion proteins, which is needed to substitute the native protein, may also lead to overestimation of protein numbers relative to endogenous expression levels. Undercounting occurs when the weak signals from FPs are missed by the localization algorithm or when FPs are not functional due to protein misfolding or incomplete maturation [10–12].

Another method of labeling employs organic fluorophores, which typically have a higher brightness and photostability than FPs, and thus have a higher probability to be successfully detected and then to be localized more accurately [13]. Organic fluorophores have not been widely used for quantification studies, however, as quantification is complicated by undercounting problems due to incomplete labeling of potential binding sites, and by overcounting problems due to reversible switching of the fluorophores and unknown stoichiometry of the fluorescent labels on the marker (e.g. antibody). These undercounting problems can only be solved in general by new advances in biochemical labeling techniques that result in a higher labeling efficiency. Instead, we focus here on addressing the overcounting problems with computational methods.

Efforts have been made in the past towards resolving the issue of overcounting with reversibly switchable fluorophores. For example, in kymograph analysis samples are prepared with sparsely distributed fluorescent markers to calibrate the fluorophore switching kinetics [8]. Similarly, a titration method was recently proposed where the concentration of markers during labeling was titrated to calibrate the number of localizations per marker [14, 15]. However, both methods are susceptible to differences in the local chemical environment in the calibration conditions that affect the switching kinetics and thus render the calibration inaccurate. Alternatively, pair correlation analysis [16, 17] does not require a separate calibration experiment, but relies on an over-simplified physical model (e.g. neglecting the effects of photobleaching). Methods addressing the short-term blinking of fluorescent proteins (e.g. [6, 8, 9]) rely on spatiotemporal clustering of localizations of the same fluorophore. This does not work for reversibly switching fluorophores as the lifetime of the long-lived dark states is much longer than the timescale on which other nearby fluorophores are activated.

In a recent paper [18] we have proposed the use of spatial frequency correlations in the reconstructed super-resolution image to estimate the average number of localizations per marker. However, in that study bleaching effects were treated in an ad hoc manner and labeling stoichiometry was not considered. Here, we present a study of how both effects can be accounted for to provide accurate quantification of localization microscopy data in terms of the number of localizations per fluorescently labeled site. Our method requires only limited calibration of the labeling stoichiometry and is applicable to common labeling techniques (e.g. antibodies). Software for estimating the number of localizations per marker with this method is freely available in the form of Matlab code at http://www.diplib.org/add-ons/.

## Results

The starting point of our analysis is a three-state switching model [19, 20] for a fluorophore consisting of an on-state, off-state and a bleached state. The on-off switching is characterized by a switching rate *k*_*sw*_ = *k*_*on*_ *k*_*off*_/(*k*_*on*_ + *k*_*off*_) and the photo-bleaching by an effective bleaching rate *k*_*bl*_. Bleaching from the on-state, as well as from the off-state, is taken into account. Therefore the effective bleaching rate *k*_*bl*_ depends on the rates of both bleaching channels. This model (see S1 Text section 2 for a derivation) gives rise to an average number of activations per fluorophore:

$$\begin{array}{c} \left\langle M(t)\rangle =M_{\infty }(1-\exp(-k_{bl}t)\right) \end{array}$$ (1)

where *M*_∞_ = *k*_*sw*_/*k*_*bl*_ is the average number of switching cycles the fluorophore undergoes before photobleaching. For small times (*k*_*bl*_ *t* ≪ 1) the statistics of on-off switching dominates the number of localizations of a single emitter, which then follows a Poisson distribution with expectation value *k*_*sw*_ *t*. For longer times (*k*_*bl*_ *t* ≫ 1) bleaching is more important and the number of localizations follows a geometric distribution with expectation value *M*_∞_.

Measurement of the bleaching rate *k*_*bl*_ from the cumulative number of localizations as a function of time is straightforward. Determination of the switching rate *k*_*sw*_, or equivalently the asymptotic number of localizations per emitter *M*_∞_, requires an additional measurement. Spatial correlation analysis with Fourier Ring Correlation [18] enables the measurement of the correlation parameter *Q*(*t*) = ⟨*M*(*M* − 1)⟩ / ⟨*M*⟩. This correlation parameter is related to the variance in *M* by *Var*(*M*) = ⟨*M*⟩ (*Q* − ⟨*M*⟩ + 1). It depends on the parameters of the three-state switching model as:

$$\begin{array}{c} Q\left(t\right)=2(M_{\infty }-1)(1-\frac{k_{bl}t}{\exp(k_{bl}t)-1})\;. \end{array}$$ (2)

Measurement of *Q*(*t*) enables the determination of *M*_∞_, as *k*_*bl*_ is already known from the fit to the cumulative number of localizations. The average number of localizations per emitter ⟨*M*(*t*)⟩ can be directly found from *k*_*bl*_ and *M*_∞_ using Eq 1. The desired density of emitters then follows from the measured density of localizations by dividing with ⟨*M*(*t*)⟩ at the final time point of the data acquisition.

**Fig 1:**
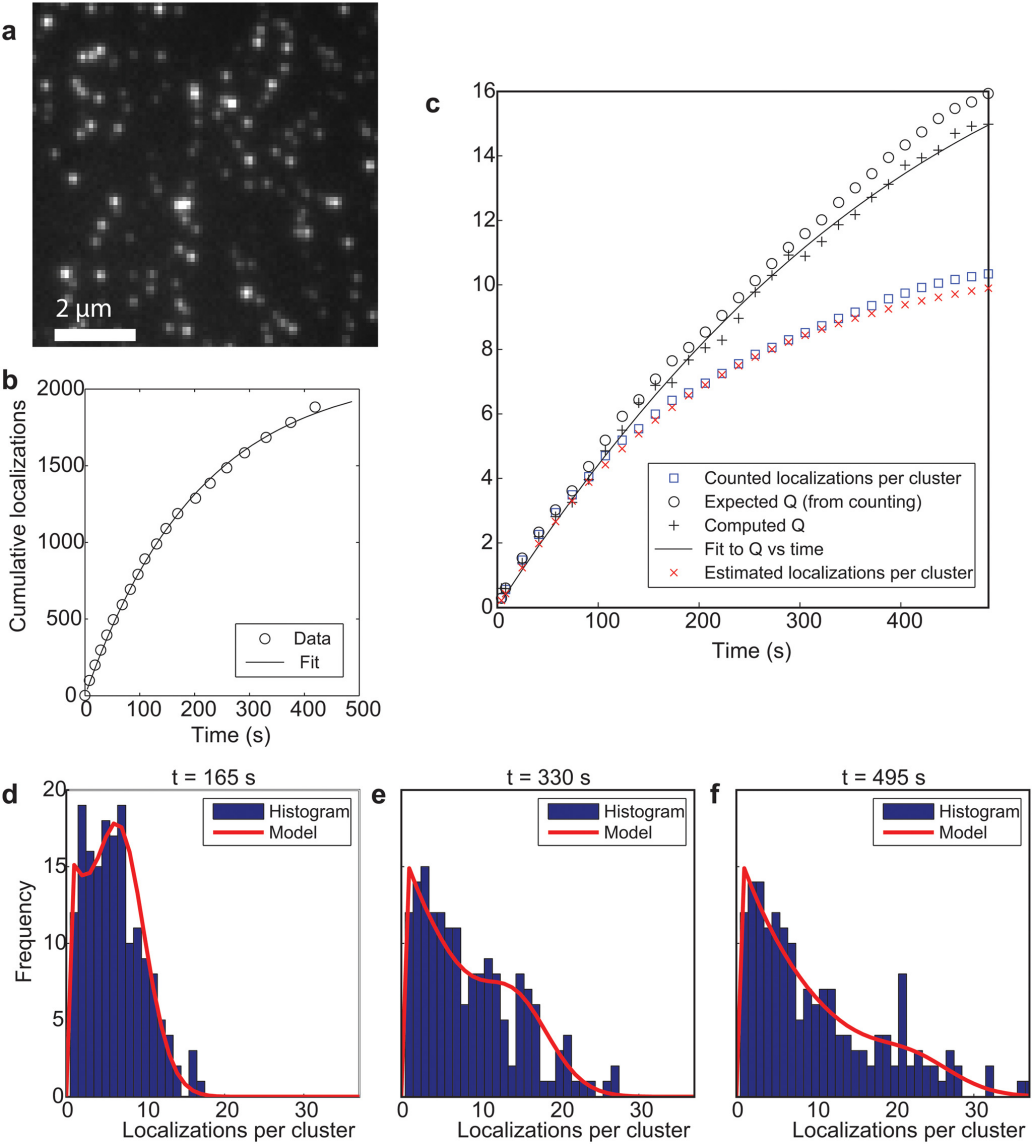
Quantitative localization microscopy with a single fluorophore per labeled site. (a) Three state model with rates. (b) Cutout of total image of sparsely distributed DNA oligomers on glass labeled with single Alexa Fluor 647 dyes showing well-isolated clusters of localizations. (c) Cumulative number of localizations and single-exponential fit. (d) Correlation parameter *Q* determined from the spatial image correlations and fit with switching model shows agreement with the ground truth value determined from the cluster analysis. The estimated value for the average number of localizations ⟨*M*(*t*)⟩ shows agreement with the ground truth value determined from the cluster statistics. (e-g) Histograms of the number of localizations accumulated per cluster and model prediction at three time points during the image acquisition.

The three-state switching model for individual fluorophores has been validated by experiments on isolated DNA oligomers labeled with single Alexa Fluor 647 dyes on a glass substrate (Fig 1a). Clearly recognizable isolated clusters of localizations provide a ground truth for the distribution of localizations per emitter. First order switching kinetics are confirmed by the observation of exponential on and off-time distributions (S1 Fig) giving *τ*_*on*_ = 26.6 ± 0.5 ms and *τ*_*off*_ = 18.0 ± 0.4 s. Fits of the cumulative number of localizations (Fig 1b) and the correlation parameter yield *k*_*bl*_ = (4.7 ± 0.1) × 10^−3^/s and *M*_∞_ = 11.0 ± 0.1. Both the correlation parameter *Q*(*t*) and the predicted number of localizations per emitter ⟨*M*(*t*)⟩, which is found with the estimated values of *k*_*bl*_ and *M*_∞_, agree with less than 10% error with the ground truth values obtained from the cluster analysis (Fig 1c). Neglecting effects of photobleaching would lead to the estimate ⟨*M*(*t*)⟩ = *Q*(*t*) which results here in an error of up to 47%. Note that the use of pair-correlation functions for counting also comes down to an alternative procedure for estimating the quantity *Q*(*t*) [17], and would thus suffer from a comparable error when photobleaching is neglected. The measured on and off-times of the clustered localizations lead to a switching rate *k*_*sw*_ = (5.6 ± 0.1) × 10^−2^/s, in reasonable agreement with the value *k*_*bl*_ *M*_∞_ = (5.2 ± 0.2) × 10^−2^/s obtained from the fit parameters above. Finally, the distribution of the number of localizations per emitter as a function of time (Fig 1d–1f) corresponds well to theory for the estimated values of *k*_*bl*_ and *M*_∞_: p = 0.67, 0.91, and 0.71, in one-sample two-sided discrete Kolmogorov-Smirnov tests at times t = 122, 243 and 365 s respectively, so no significant difference was found at a 0.05 significance level.

**Fig 2:**
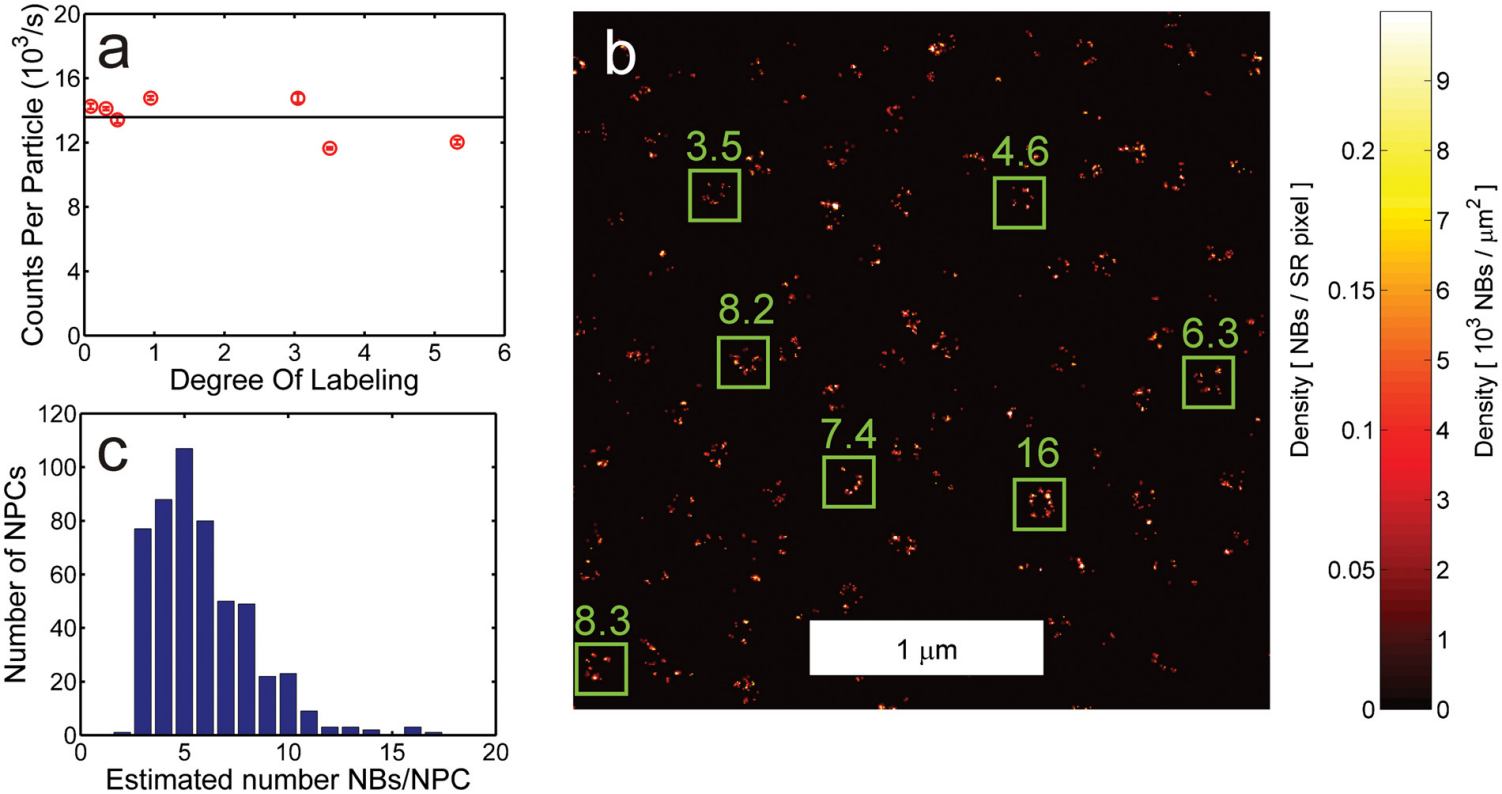
Quantitative localization microscopy of NB-labeled Seh1 in the NPC. (a) FCS-analysis of NB stoichiometry indicating there is a single fluorophore per NB. (b) Cutout of quantitative localization microscopy image of NB-labeled Seh1 in the NPC (*k*_*bl*_ = 4.8 × 10^−3^/s and *M*_∞_ = 5.0). The numbers at the green boxes indicate the estimated number of NBs within the box. (c) Histogram of the estimated number of NBs per NPC.

We applied our method to images of the Seh1 protein, a component of the Nuclear Pore Complex (NPC) [21] tagged with mEGFP and labeled with anti-GFP nanobodies (NBs) conjugated to Alexa Fluor 647 fluorophores. The degree of labeling (DOL) and average brightness of the NBs were characterized with absorption spectroscopy and Fluorescence Correlation Spectroscopy (FCS), respectively. This revealed that only one emitter per NB contributes to fluorescence imaging due to quenching effects (Fig 2a), implying that counting the number of fluorophores is equivalent to counting the number of NBs. The resulting quantitative localization microscopy image is shown in Fig 2b. We found that the estimated number of NBs bound per NPC varies between 3 and 17 (Fig 2c). This indicates that the labeling efficiency was relatively low, given the eightfold symmetry of the NPC and given that recent stoichiometry data point to up to 32 Seh1 copies per NPC [22, 23].

In commonly used antibody labeling schemes there are *S* > 1 fluorescent molecules per labeled site (e.g. antibody). The three-state switching model can be expanded to incorporate this labeling stoichiometry (see S1 Text section 3) from which we obtain an average number of activations per labeled site ⟨*M*⟩ and a correlation parameter *Q*:

$$\begin{array}{c} \langle M(t)\rangle =\langle S\rangle M_{\infty }\;(1-\exp(-k_{bl}t))\;, \end{array}$$ (3)

$$\begin{array}{c} Q(t)=2M_{\infty }(1-\frac{k_{bl}t}{\exp(k_{bl}t)-1})+\mu M_{\infty }\;(1-\exp(-k_{bl}t))\;, \end{array}$$ (4)

where the average number of emitters per site ⟨*S*⟩ and the stoichiometry parameter *μ* = ⟨*S*(*S* − 1)⟩ / ⟨*S*⟩ are novel parameters entering the description. The averages here are understood to be averages over the distribution of labeled sites (sites with *S* ≥ 1). When each labeled site has only one emitter we have ⟨*S*⟩ = 1 and *μ* = 0, and we retrieve the previously considered case of Eqs 1 and 2. Expressions for the average number of emitters per site ⟨*S*⟩ and the stoichiometry parameter *μ* can be derived from models for the labeling stoichiometry (S1 Text section 3).

Primary antibody labeling may be described by Poisson statistics for weakly interacting fluorophores. Then the DOL revealed by absorption spectroscopy corresponds to the Poisson rate of the labeling process. It follows that the average number of emitters per site ⟨*S*⟩ = DOL/ [1 − exp(−DOL)] and the stoichiometry parameter *μ* = DOL. Quenching (usually attributed to dye aggregation [24–26]), invalidates the assumption of weakly interacting fluorophores for larger DOL values [14] and a separate calibration of both the average number of emitters per site ⟨*S*⟩ and the stoichiometry parameter *μ* (but not of the switching and bleaching rates *k*_*sw*_ and *k*_*bl*_) is then necessary. The case of secondary antibody labeling is even more complicated as now the stoichiometry of secondary to primary antibodies is relevant in addition to the stoichiometry of emitters on the secondary antibodies (S1 Text section 3). Generally, prior knowledge on the labeling via a calibration of the average number of emitters per site ⟨*S*⟩ and the stoichiometry parameter *μ* is needed to compute the average number of localizations per labeled site ⟨*M*(*t*)⟩ from the cumulative number of localizations and the correlation parameter *Q*(*t*).

**Fig 3:**
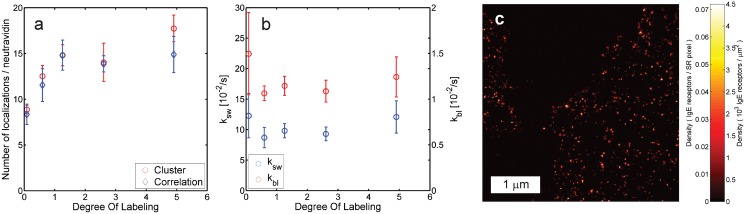
Quantitative localization microscopy with multiple emitters per labeled site. (a) Number of localizations per neutravidin tetramer as a function of DOL as estimated from the image correlations and the ground truth values from cluster analysis, showing good agreement. (b) Fitted bleach rate *k*_*bl*_ and switching rate *k*_*sw*_ = *M*_∞_ *k*_*bl*_ as a function of DOL values for the same data, indicating independent activation and bleaching per label. Error bars indicate the standard deviation among samples at the same DOL. (c) Image of IgE receptors on the membrane of RBL cells labeled with primary antibodies with a DOL of 1.5 (*k*_*bl*_ = 9.1 × 10^−3^/s and *M*_∞_ = 2.3).

We validated the approach for estimating the number of localizations per site with multiple fluorophores per site using a control sample of sparsely distributed neutravidin tetramers on glass labeled with varying numbers of Alexa Fluor 647 fluorophores. The labeling stoichiometry parameters were determined from FCS brightness measurements and from the brightness statistics of single neutravidin tetramers in the first frames of the sparse control samples (S2 Fig panels a and b). The values obtained with the latter method were applied to estimate the number of localizations per neutravidin tetramer with a Root Mean Square Error (RMSE) of 17% of the ground truth number, which was established by cluster analysis (Fig 3a). This result appears to be robust against errors in the calibration of the stoichiometry parameter *μ*, as variations in this parameter on the order of unity change the result by 10% or less (S3 Fig). The estimated switching model parameters *M*_∞_ and *k*_*bl*_ do not vary significantly with DOL (Fig 3f), suggesting independent switching and bleaching of the detected, non-quenched emitters (see also S2 Fig panel c). The remaining quenched emitters that do contribute to the measured DOL in absorption spectroscopy do not appear to contribute to the localizations (S2 Fig panels a and d). Additional validations of our method for multiple fluorophores per site are shown in S4 and S5 Figs.

Next, we applied our counting method to images of Immunoglobulin E (IgE) receptors in fixed Rat Basophilic Leukemia (RBL) cells labeled with IgE conjugated to Alexa Fluor 647 (Fig 3c). The data were analyzed assuming a stoichiometry parameter *μ* = DOL and an average number of emitters per site ⟨*S*⟩ = DOL/ [1 − exp(−DOL)], where the measured DOL = 1.5 was low enough to neglect possible quenching effects. The density of receptors on the membrane was estimated as 81*μ*m^-2^. This is on the same order as e.g. Espinoza et al. [27], where on average 64 ± 32*μ*m^-2^ were obtained in TEM images (252 ± 123 receptors per field of view of (2266 nm)^2^ with a labeling efficiency of 0.8 ± 0.1). Densities may vary substantially with cell incubation times and between cell types though, implying that more precise values cannot be specified.

Care must be taken when applying our analysis to samples that have markers with mutual distances well below the localization precision due to high labeling densities or clustering. Effectively these markers would be seen as a single labeled site by the current correlation analysis algorithm, which causes overestimation of the number of localizations per marker. S1 Text section 4 provides estimates for the labeling densities above which problems are to be expected. As a rule of thumb, problems ar expected when the density is higher than 1/*σ*_*m*_ (for filaments) or $1/2\sigma_{m}^{2}$ (for punctate clusters), where *σ*_*m*_ is the average localization precision.

**Fig 4:**
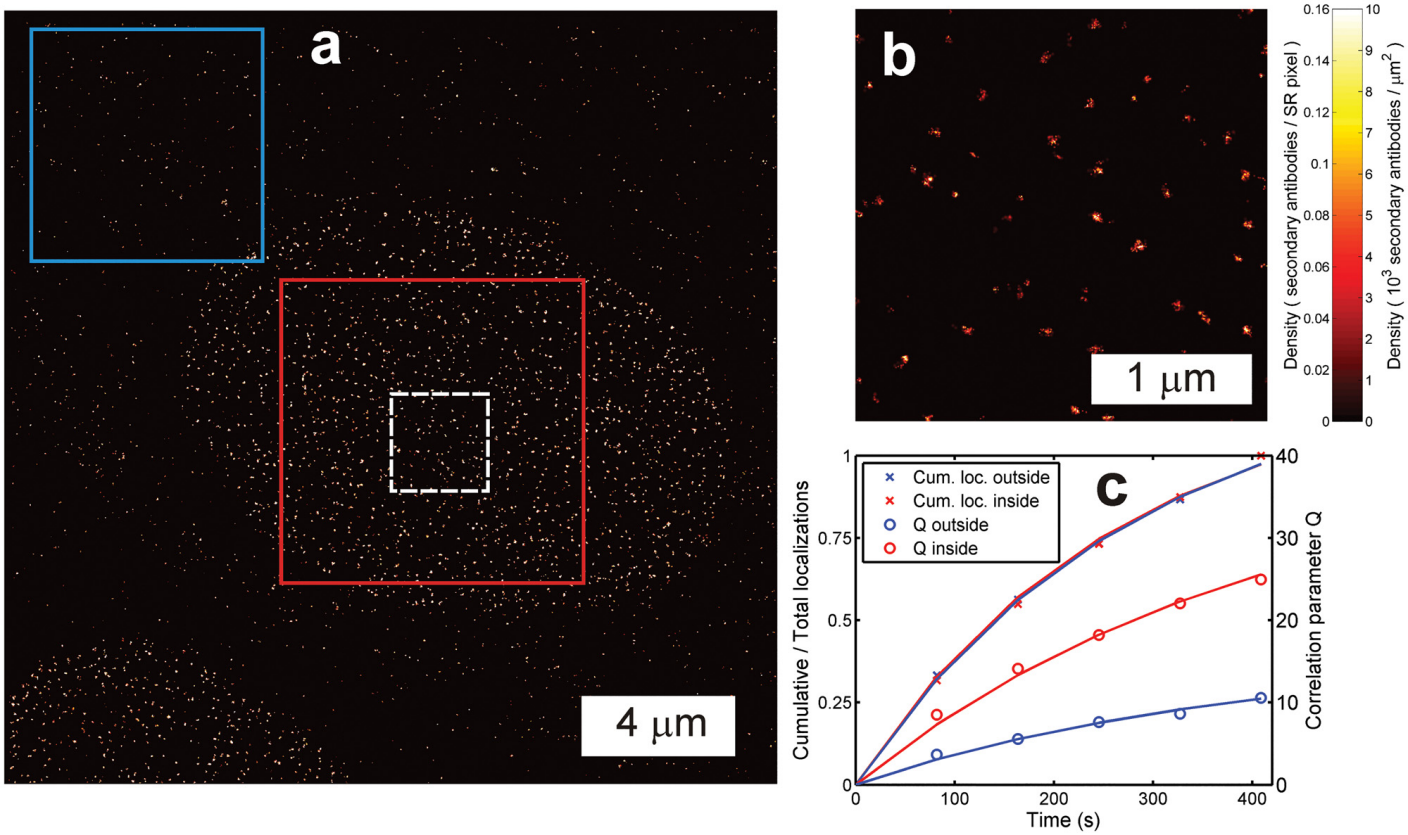
Quantitative localization microscopy with heterogeneous labeling density. (a) Overview image (pixel size 10 nm, clipped for visibility) and (b) zoomed inset (pixel size 4 nm) of the dashed white box in (a) of secondary antibody-Alexa Fluor 647 labeled Nup153 protein of the NPC in the nuclear membrane with non-specifically bound (secondary) antibodies outside the nuclear membrane region. (c) The correlation parameter Q for the region inside the nuclear membrane (red box) is higher than outside (blue box) due to the tight clustering of the secondary antibodies labeling the Nup153 proteins. The relative number of accumulated localizations at each time point is similar, indicating that the bleaching behavior is similar and the sources of the localizations are identical in both regions.

An experimental approach to verify that counting results are not affected by high density artefacts is to compare them with the results that are obtained by computing the correlation parameter *Q*(*t*) in regions of relatively low labeling density. We have analyzed an image of secondary antibody-Alexa Fluor 647 labeled Nup153 protein of the NPC in this way (Fig 4). The densely labeled region with NPCs inside the nuclear membrane gives rise to a correlation parameter *Q*(*t*) that is about 2.4 times higher than for the region with non-specifically bound antibodies outside the nuclear membrane. This shows that the clustered antibodies inside the nucleus appear as a single site for the estimation of *Q*(*t*). However, the relative rate with which localizations are accumulated is similar, indicating similar bleaching behavior and identical fluorophores in both regions. A fit to the correlation parameter *Q*(*t*) for the outside region gives *k*_*bl*_ = 3.8 × 10^−3^/s and *M*_∞_ = 5.5, under the assumption that the outside region labeling entities are secondary antibodies, and using the calibrated fluorophore to secondary antibody DOL equal to 1.2. Applying these values in a fit of the correlation parameter *Q*(*t*) for the region inside the nuclear membrane gives approximately 2.7 secondary antibodies per NPC on average, which agrees with the ratio of 2.4 between the localizations per NPC and the localizations per non-specifically bound antibody outside the nucleus. The NPCs are likely to have multiple primary antibodies, because this would explain the difference in the spread of localizations of NPCs (16 nm) and localizations of the non-specifically bound antibodies outside the nuclear membrane region (11 nm).

We also verified that the counting results in Fig 2 are not affected by high density artefacts by computing the correlation parameter *Q*(*t*) in a region outside the nucleus with similar bleaching behavior. A fit to *Q*(*t*) returned *k*_*bl*_ = 5.3 × 10^−3^/s and *M*_∞_ = 4.5 (compared with *k*_*bl*_ = 5.3 × 10^−3^/s and *M*_∞_ = 5.0 inside the nucleus), which showed that the estimation of *Q*(*t*) was not substantially affected by clustering of Seh1 in these data.

## Discussion

The switching model assumes constant and uniform rates. Accordingly, all data was acquired under conditions where the excitation and activation light intensities did not vary spatially across the sample, nor change as a function of time during the experiment. To adapt the method for experiments in which the switching rate is varied, the illumination intensities should be recorded over time and included in a generalization of the switching model that includes time dependent switching rates. The method has been demonstrated on Alexa Fluor 647 dyes, but applies to any fluorophore that can effectively be described by the three-state switching model. Such a description becomes problematic for the existence of multiple long lived dark states with lifetimes on the same order of magnitude [20]. This would require a more substantial modification of the theory, in which the three-state model is expanded with one or more additional states and two or more additional rates between the states. Subsequently, the average number of localizations per labeled site ⟨*M*(*t*)⟩ needs to be derived and expressed in a form that only depends on parameters that can be obtained from fits to the cumulative number of localizations and to the correlation parameter *Q*(*t*). Finally we note that fluorophore activation events that are missed by the localization algorithm, so-called false negative localizations, do not affect the accuracy of the method by more than 5 to 10% (see S1 Text, section 3).

The analyses for the data presented in Figs 1 and 3a showed that overcounting errors on the order of 50% occur when neglecting reversible switching of the fluorophores and unknown stoichiometry of the fluorescent labels, as is typically done for example in pair-correlation analysis [17]. As we noted before, the latter represents an alternative approach for estimating the spurious correlation parameter *Q*(*t*), and could therefore be corrected for overcounting similar to how we treat the estimate for *Q*(*t*) from the FRC. However, the pair-correlation analysis does require a parametric model for the correlations in the spatial distribution of the labeled sites, unlike the FRC approach. In-vitro calibration of fluorophore switching and bleaching rates for counting purposes may be susceptible to differences in the chemical environment of the fluorophores. Comparing the estimated rates for Neutravidin in Fig 3 (on glass) and S4 Fig (in a cell) indicates that differences in these rates of a factor 2 to 3 may occur, which would result in similar differences in the estimated number of localizations per site.

In summary, we have developed a method for estimating the number of localizations per fluorescently labeled site in order to resolve overcounting problems with reversibly switchable emitters. For labeling entities with single fluorophores the method can be used directly on the localization data. Otherwise the method requires only a one-time calibration of the number of fluorophores per label as an additional input, which can be used for all subsequent uses of that label. With spatial resolution approaching the molecular scale, this will expand the possibilities of researchers to address questions about the molecular stoichiometry and spatial organization of protein complexes. This is essential to establish localization microscopy as a method which may be used to not just observe the nanoscale “shape” of biological structures, but also to obtain quantitative information about their composition.

## Materials and Methods

### Experimental materials and methods

**Preparation of fluorescent DNA oligonucleotides** To characterize the on-off switching kinetics of single reversibly switchable fluorescent molecules, a single Alexa Fluor 647 fluorophore was conjugated to the end of a double stranded DNA (dsDNA) construct, and the construct was immobilized on a glass surface for single-molecule imaging. DNA constructs were labeled as previously described [19, 28]. Briefly, PAGE purified DNA oligos (30 base pairs in length) with biotin and/or amine modifications at the ends were obtained from Eurofins Operon. Amine-modified oligos were labeled post-synthesis with amine reactive Alexa Fluor 647 (Life Technologies, A20006) following the manufacturer’s protocol. Dye-labeled oligos were purified using reverse-phase HPLC. Complimentary strands of DNA, one with a biotin label and the other with a fluorescent label, were annealed to form fluorescent biotinylated dsDNA. Annealing was carried out by mixing equimolar amounts of the two complimentary strands in 10mM Tris-Cl (pH 7.5), 50mM NaCl, heating for 60s at 90°C, and cooling to room temperature during ∼1 hr.

**Preparation of fluorescent Secondary Antibodies, Nanobodies, and Neutravidin** Donkey anti-mouse secondary antibodies (Jackson ImmunoResearch # 715-005-150), anti-GFP camelid antibody fragments (a.k.a. Nanobodies, Chromotek, GT-250), and Neutravidin tetramers (Life Technologies, A2666) were labeled with amine-reactive Alexa Fluor 647 according to the manufacturer’s protocol. Briefly, unlabeled antibodies, nanobodies, or neutravidin were mixed with amine reactive dye in a sodium bicarbonate buffer (0.1 M, pH 8.5), and the labeling reaction was left to proceed at room temperature for 30 min. The labeled product was separated from unreacted dye by running the reaction mixture over a gel filtration column (Illustra NAP-5 column, GE Healthcare), and eluting in PBS. The labeled product was stored at 4°C in PBS. The degree of labeling (DOL) of the antibodies, nanobodies, or neutravidin was measured using a UV/Vis spectrophotometer. The DOL was adjusted by varying the amount of dye that was added to the reaction.

**FCS characterization of fluorescent Nanobodies, Neutravidin, and Secondary Antibodies** The fluorescence lifetime and brightness per particle of fluorescent antibodies, nanobodies, and neutravidin were measured using a commercial FCS spectrometer (Evotec FCS plus spectrometer, Evotec Technologies, Hamburg, Germany). This instrument has been described in detail previously [29]. Samples were diluted in PBS or in MEA imaging buffer (see below) and loaded into 96-well plates. The sample was illuminated with a pulsed 633nm laser diode (Picoquant) and imaged using an Olympus 60X 1.2NA water immersion objective and a confocal detection scheme. Fluorescence intensity traces were recorded and analyzed using the Evotec FCS++ analysis software. This yielded measurements of fluorescence brightness per particle and fluorescence lifetime for each sample.

**Single molecule imaging of immobilized DNA and Neutravidin** The labeled dsDNA was immobilized on glass coverslips via a biotin-streptavidin linkage. A biotinylated BSA solution (1.0 mg/mL, Sigma Aldrich) was first added to the coverslip, followed by 0.25 mg/mL streptavidin (Life Technologies), and finally the DNA sample at a low concentration (∼30 pM) in order to obtain a low surface density of DNA molecules such that individual molecules were well separated and optically resolvable from each other. The surface was rinsed with 10mM Tris-Cl (pH 7.5), 50mM NaCl solution prior to the addition of each reagent. MEA imaging buffer, described below, was added to the sample prior to imaging. Single molecule Neutravidin samples were prepared in a similar way. A biotinylated BSA solution was first added to the coverslip, followed by rinsing with Tris buffer, and then the neutravidin sample was added at a low concentration (∼50 pM). Following a second rinsing step, the surface density of labeled neutravidin molecules was low enough such that individual molecules were well separated and optically resolvable from each other. MEA imaging buffer, described below, was added to the sample prior to imaging.

**Imaging buffer** All imaging experiments, including measurements of single molecule switching and STORM imaging, were carried out in MEA imaging buffer as previously described [19, 28]. The imaging buffer consists of 50mM Tris-Cl (pH 8.0), 10mM NaCl, 10% Glucose (w/v), 10mM *β*-mercaptoethylamine (pH 8.5, Sigma, 30070), and 1 of an enzymatic oxygen scavenger system stock solution. The oxygen scavenging system was added to the buffer immediately before use. The oxygen scavenger stock solution was prepared by mixing glucose oxidase powder (10 mg, Sigma, G2133) with catalase (50 *μ*L, 20 mg/mL, Sigma, C30) in PBS (200 *μ*L), and centrifuging the mixture at 13.000 rpm for 1 minute.

**Fluorescent staining of cultured cells** For experiments involving actin imaging, Vero cells were plated on coverslips and fixed in 4% paraformaldehyde for 10 minutes at room temperature. The cells were permeabilized in 0.1% triton in PBS for 5 minutes, and then washed 3 times with blocking buffer (2% BSA in PBS) for 5 minutes. Cells were then labeled with biotin-xx phalloidin (Life Technologies, B7474) at 1:50 dilution in blocking buffer for 1 hour. Cells were rinsed with PBS 3 times for 5 minutes, and then labeled with fluorescent neutravidin (DOL 1.28) at a high dilution in blocking buffer for 1 hour. The cells were rinsed in PBS before mounting in imaging buffer and imaging.

For experiments involving tubulin imaging, Ptk2 cells were fixed with ice-cold methanol for 4 minutes, before washing 3 times for 5 minutes in blocking buffer. Cells were labeled with mouse anti-tubulin primary antibodies (Sigma T6074) at 1:100 dilution in PBS for 1 hour at room temperature, followed by 3 washes for 5 minutes in blocking buffer. The secondary antibody was added at a high dilution in blocking buffer for 1 hour. The sample was rinsed in PBS before mounting in imaging buffer and imaging.

For fluorescent imaging of Nup153, Vero cells were fixed, permeabilized, and blocked as described above for the case of actin imaging. Cells were labeled with mouse anti-Nup153 primary antibodies (Abcam ab24700) at 1:100 dilution in PBS for 1 hour at room temperature, followed by 3 washes for 5 minutes in blocking buffer. The sample was inclubated with the secondary antibody in blocking buffer for 1 hour. The sample was rinsed in PBS before mounting in imaging buffer and imaging.

For the NPC staining of Seh1, a Hela Kyoto cell line stably expressing an siRNA-resistant version of the human Seh1 transcript tagged with mEGFP was established by selection of cells transfected with pmEGFP-Seh1-s37879res [30] with 1 mg/mL Geneticin (Life Technologies). To increase the degree of replacement of the endogenous protein with the mEGFP-tagged version, the cells were repeatedly transfected every 48 hours over the course of 12 days with Silencer Select siRNA s37879 against Seh1 (Life Technologies) by solid phase transfection on siRNA-coated 24-well plates (for details on the coating procedure see Szymborska et al. [30]). After knock down, the cells were transferred onto cover slips, allowed to attach and processed for staining with Alexa Fluor 647-coupled anti-GFP nanobody as described before [30]. For imaging we chose cells with low cytoplasmic GFP signal and excluded cells with aberrant nuclear shape.

**Microscope** All imaging measurements were performed using a custom built inverted fluorescence microscope, similar to that described previously [31]. To summarize, an inverted fluorescence microscope stand (Olympus IX71) was fitted with a 100X oil-immersion objective lens (Olympus, UPLANSAPO100XO) which enabled efficient detection of single fluorophores. A custom-built focus lock system based on the reflection of an infra-red laser from the sample was used to maintain sample focus during all measurements. For STORM imaging, photo-switchable Alexa Fluor 647 was excited using 642 nm light, and in some measurements the sample was also exposed to 405 nm light to increase the activation rate of switching. A solid-state diode laser (Oxxius) was used to generate 405 nm light, and a fiber laser (MPB Communications, 2RU-VFL-P-1500-642) was used to generate 642 nm light. The laser illumination was configured such that the illumination angle could be varied between an epi-illumination geometry and a total internal reflection (TIRF) illumination mode. For STORM data acquisition, the sample was illuminated with oblique illumination (not TIRF) for reduced background signal. Fluorescence emission of Alexa Fluor 647 was filtered using a dichroic mirror (Chroma, Z660DCXRU) and a bandpass emission filter (Chroma, ET700/75). Fluorescence was detected using an EMCCD camera (Andor Technology, Ixon DU897).

**Imaging of IgE** RBL cells were seeded on aminosilane coverslips in Lab-Tek eight-well chambers (Nunc). The cells were then incubated for 60 min. at 37°C with 1 *μ*g/mL Alexa Fluor 647-conjugated IgE with a dye/antibody ratio of 1.5. Subsequently, cells were rinsed thrice for 5 min. in Phosphate Buffered Saline (PBS). Then, the cells were fixed in 4.0% (wt/vol) paraformaldehyde and 0.2% glutaraldehyde in phosphate-buffered saline (PBS) for 60 min at room temperature, after which they were rinsed twice for 5 min. with 10 mM Tris and stored in PBS for imaging. Right before imaging, the cells were immersed in an imaging buffer consisting of 450 *μ*L 10% (w/v) glucose in 50 mM Tris, 10 mM NaCl, pH 8.5; 50 *μ*L oxygen scavenger buffer [14040U catalase (C9322-1G, Sigma Aldrich), 1688U glucose oxidase (G2133-50KU, Sigma Aldrich) in 50 mM Tris, 10 mM NaCl, pH 8.5; 5 μL 1M mercaptoethylamine (MEA), pH 8.5. The IgE samples were imaged with an epifluorescence microscope setup, consisting of an inverted microscope (IX71, Olympus), a 1.45-NA TIRF objective (U-APO 150X NA 1.45, Olympus), a 637-nm diode laser (HL63133DG, ThorLabs, with home built collimation optics) and an EMCCD camera (iXon 897, Andor) with EM gain set to 200. Samples were mounted into a 3D piezo stage (Nano-LPS100, Mad City Labs). For sample illumination and emission, a quad-band dichroic and emission filter set was used (FF01-446/523/600/677-25, Semrock). Images were taken in a TIRF configuration at 57 frames per second for 33,000 frames.

### Data analysis methods

**Localization analysis** Identification of regions of interest and estimation of the fluorophores’ position followed established methods [28, 32, 33]. Localizations corresponding to the same activation event were subsequently combined by grouping spatially nearby localizations in subsequent frames into single localization events. ‘Nearby’ is defined here as having a distance less than five times the sum of the localization uncertainty of the two to-be merged localization events. The tolerance in the distances was chosen relatively high because the risk of accidentally combining localizations from different nearby molecules was low in view of the sparsity of the image. The center position of the grouped localizations was determined as the weighted average of the localizations with the inverse of the localization variances as weights. Localizations were filtered based on the photon count per localization before and after combining localizations per activation event, photons per activation event, activation event duration and fitted PSF full width at half maximum. An overview of filter values is shown in Table 1. Photon count thresholds were chosen relatively high to filter out localizations due to sample contaminations for obtaining accurate results in the cluster analyses (S6 Fig). Localizations were finally corrected for lateral stage drift using frame-by-frame cross-correlation, as documented elsewhere [28, 34].

**Table 1:** Parameters used for filtering localization events. Localizations were filtered for the minimum number of photons per event before grouping, minimum number of photons per event after grouping, the maximum duration of the event after grouping, and the maximum width (FWHM) of the Gaussian fitted to the spot.

**Dataset**	**Photons before**	**Photons after**	**Duration (frames)**	**Width (nm)**
DNA oligomers	500	5,000	100	377
Nuclear Pore Complex	1,200	2,000	20	283
Neutravidin	1,200	3,000	20	283
Tubulin	500	5,000	100	377

**Estimating the correlation parameter Q** The first step towards estimating the number of localizations per marker consists of estimating the spurious correlation parameter *Q* at various points in time; typically 30 time points were used. The first steps of this estimation of *Q* were the same as done previously [18] and culminate in the determination of the numerator *ν*(*q*) of the Fourier Ring Correlation (FRC) for spatial frequencies *q* = 1/*L*, 2/*L*, … (*L* is the size of the field of view). Briefly, the full set of estimated fluorophore positions is divided into two independent subsets. This yields two sub-images $f_{1}(\vec{r})$ and $f_{2}(\vec{r})$, where $\vec{r}$ denotes the spatial coordinates. Subsequently the Fourier transforms of those images, ${\hat{f}}_{1}\left(\vec{q}\right)$ and ${\hat{f}}_{2}\left(\vec{q}\right)$ respectively, are computed. The statistical correlation between those Fourier transforms is then evaluated over pixels on the perimeter of circles in Fourier space with radius *q*:

$$\begin{array}{c} \nu (q)=\frac{1}{2\pi qL}\sum_{\vec{q}\in \text{circle}}{\hat{f}}_{1}\;(\vec{q}){\hat{f}}_{2}\;{\left(\vec{q}\right)}^{*}, \end{array}$$ (5)

At high spatial frequencies *q*, *ν*(*q*) is dominated by spurious correlations due to multiple localizations of the same site. Thus, the spurious correlation parameter *Q* is computed by fitting *ν*(*q*) with the following model function [18]:

$$\begin{array}{c} H(q;\sigma_{m},\Delta \sigma )=\frac{1}{\sqrt{1+8\pi^{2}\Delta \sigma^{2}q^{2}}}\exp(\frac{4\pi^{2}\sigma_{m}^{2}q^{2}}{1+8\pi^{2}\Delta \sigma^{2}q^{2}})\;\text{sinc}{\left(q\right)}^{2}. \end{array}$$ (6)

This function describes the theoretical decay of the spurious correlations, assuming that the uncertainties *σ* of localizations follow a normal distributed with unknown mean *σ*_*m*_ and standard deviation Δ*σ*.

The actual fit to *ν*(*q*) is obtained using a novel method which involves the minimization of the cost function:

$$\begin{array}{c} C_{\nu }(Q,\sigma_{m},\Delta \sigma )=-\sum_{q}\exp(-\frac{{\left(\nu \left(q\right)-QH\left(q;\sigma_{m},\Delta \sigma \right)\right)}^{2}}{d^{2}Q^{2}H{\left(q;\sigma_{m},\Delta \sigma \right)}^{2}}) \end{array}$$ (7)

where *d* was chosen to be 0.1. The rationale behind this cost function is that it promotes parameters for which *ν*(*q*)/*H*(*q*) is constant for a large range of spatial frequencies. This objective was used in our previous work as a requirement for the manually provided parameters *σ*_*m*_ and Δ*σ* [18]. The search for parameters *Q*, *σ*_*m*_ and Δ*σ* that minimize *C*_*ν*_ was done with the Nelder-Mead simplex algorithm. This algorithm was initialized two times, where the starting values for the second optimization were randomly perturbed with respect to the first.

For each time *t*, this procedure of dividing localizations into subsets, computing *ν*(*q*) and fitting it to obtain values for *Q*, *σ*_*m*_ and Δ*σ* was repeated five or ten times with randomly perturbed initial values for *σ*_*m*_ and Δ*σ*. The median of the different estimates of *Q*(*t*) at each time *t* was then taken to obtain a robust estimation result for *Q*(*t*).

**Estimating the number of localizations per labeled site** After the correlation parameter *Q*(*t*) is obtained at various time points *t*, the next step in estimating the number of localizations per labeled site *M* involves a simultaneous model fit to *Q*(*t*) and the cumulative number of localizations *N*(*t*). This is achieved by minimizing the cost function:

$$\begin{array}{c} C_{Q}(M_{\infty },k_{bl},N_{\infty })=\sum_{t}\;\{\frac{{\left(N\left(t\right)-N_{model}\left(t\right)\right)}^{2}}{N_{model}\left(t\right)N_{model}\left(t_{end}\right)}-\frac{{\left(Q\left(t\right)-Q_{model}\left(t\right)\right)}^{2}}{Q_{model}{\left(t_{end}\right)}^{2}}\}\; \end{array}$$ (8)

where the sum runs over all times *t* for which the spurious correlation parameter was estimated, *t*_*end*_ is the total acquisition time, and:

$$\begin{array}{c} N_{model}(t)=N_{\infty }\;(1-\exp(-k_{bl}t)) \end{array}$$ (9)

$$\begin{array}{c} Q_{model}(t)=2M_{\infty }\;(1-\frac{k_{bl}t}{\exp(k_{bl}t)-1})+\mu M_{\infty }\;(1-\exp(-k_{bl}t)). \end{array}$$ (10)

Optimizing *C*_*Q*_ was again performed using the Nelder-Mead simplex algorithm. The parameter *μ* was a separate manual input for the optimization for the purpose of this work, obtained from a calibration detailed below. The fitted values *M*_∞_ and *k*_*bl*_ are used to obtain the final estimate:

$$\begin{array}{c} M\left(t\right)=\langle S\rangle \;M_{\infty }\;(1-\exp(-k_{bl}t))\; \end{array}$$ (11)

where the average number of emitters per labeled site ⟨*S*⟩ was obtained from the same calibration as *μ*. Potentially, *μ* could be obtained from a fit of *Q*(*t*), completely eliminating the need for calibration experiments. It turned out, however, that for the datasets we considered this could not be done reliably, possibly due to residual errors in extracting *Q*(*t*) from the data or flaws in the switching model.

**Calibration of the labeling stoichiometry** The stoichiometry parameter *μ* was calibrated for the Neutravidin data as follows. The localizations obtained for the datasets of Neutravidin on glass were clustered as described below. Subsequently, clusters were discarded if there was another cluster within a square region of 7 CCD pixels around each of them. For the remaining clusters, the site was localized in the first frame of the raw sequence to accurately determine the number of signal photons *B* of the site in that frame. If we assume that each fluorophore is active during the entire first frame, then computing the average and variance of the brightness over the found clusters provides the following equalities:

$$\begin{array}{c} \left\langle B\rangle =\langle B_{single}\rangle \;\langle S\right\rangle \end{array}$$ (12)

$$\begin{array}{c} \frac{\left\langle B^{2}\right\rangle }{\left\langle B\right\rangle }=\frac{\left\langle B_{single}^{2}\right\rangle }{\left\langle B_{single}\right\rangle }+\langle B_{single}\rangle \;\mu \end{array}$$ (13)

Here, *B*_*single*_ is the brightness of a single emitter. For small DOL values it is assumed that the labeling is described by Poisson statistics giving *μ* ≈ DOL and ⟨*S*⟩ ≈ DOL/ (1 − exp(−DOL)). A linear fit on the data points for ⟨*B*⟩ with DOL < 2 gives values for ⟨*B*_*single*_⟩, which are subsequently used to find values for ⟨*S*⟩ for all for all DOL-values. Similarly, a linear fit on the data points for ⟨*B* ^2^⟩ / ⟨*B*⟩ for DOL < 2 is used to find the parameters needed to compute *μ* for all DOL-values. It appears that the value for ⟨*B*_*single*_⟩ fitted from Eq 13 is a factor 1.4 higher than the value fitted from Eq 12, possibly due to a bias in the clustering procedure or due to a breakdown of the Poisson assumption. Bleaching in the initial switching-off phase of the data acquisition may introduce a small bias in the calibration procedure towards higher values of ⟨*S*⟩ and *μ* (relative error at most about 1/*M*_∞_).

In an alternative calibration approach, the markers were analyzed in solution with Fluorescence Correlation Spectroscopy (FCS). The brightness per marker can be analyzed to find values for ⟨*S*⟩ just as done for the cluster brightness analysis. Values for the stoichiometry parameter *μ* are found by inverting the Poisson relation ⟨*S*⟩ = *μ*/((1 − exp(−*μ*))), which gives rise to biases in the quenching regime DOL > 2.

**Cluster analysis** The ground truth for the distribution of the number of emitters per labeled site for the data of Fig 1c (DNA oligomers) and Fig 3a (neutravidin tetramers) was established from the following steps. First, an image was created in which each localization was rendered as a Gaussian blob with a maximum of 1 and a standard deviation equal to the localization uncertainty obtained from the localization algorithm. The pixel size in these images was 8 nm. Subsequently, these images were thresholded at a value of 10^−3^, 8-connected regions of nonzero pixels were identified and the localizations in these regions were assigned to clusters. For each cluster, the center position was determined using weighted-least squares estimation. The sum of squared Mahalanobis distances from the localizations to their cluster centers was then computed and clusters where this sum was significantly larger than expected for a sum of Gaussian localization errors (at statistical significance level of 10^−3^) were discarded for further analysis. Finally, clusters without localizations before a specified time threshold were discarded on the suspicion that they were due to sample contaminations rather than fluorophores (S3 Fig); for the DNA oligomer data the threshold was at 10,000 frames, for the Neutravidin data at the 95 percentile value of the times between localizations in clusters. The remaining clusters of localizations were considered to be localizations of the same labeled site.

For the somewhat denser tubulin samples a different clustering method was found more suitable, based on nearest-neighbor linking. Localization events are considered as belonging to the same cluster if their relative distance $|\vec{r}|<R\approx 2\sigma$, with *σ* the localization uncertainty. The likelihood of localizing an emitter at position $\vec{r}$ from the true emitter position is a Gaussian in x and y with standard deviation *σ*. Therefore the likelihood of two localizations of the same emitters at relative position $\vec{r}$ is a Gaussian in x and y with standard deviation $\sqrt{2}\sigma$, as follows by convolution of the two individual Gaussian likelihood functions. It follows then that the likelihood of two localizations of the same emitters at relative distance $|\vec{r}|<R$ is:

$$\begin{array}{c} P(|\vec{r}|<R)=1-\exp(-R^{2}/4\sigma^{2}) \end{array}$$ (14)

so, for *R* = 2*σ* we find a likelihood for correctly linking two localizations of the same emitter $P(|\vec{r}|<R)=1-1/e=0.63$. As typically each cluster consists of ∼ 10 localizations most clusters will be correctly detected. After initial nearest-neighbor linking clusters with less than 2 or more than 50 localizations are filtered out. Subsequently, the distribution of localization uncertainties of the detected clusters is evaluated. Clusters with a localization uncertainty *σ* > *σ*_*m*_ + 2Δ*σ*, with *σ*_*m*_ and Δ*σ* the mean and standard deviation of the distribution of localization uncertainties, are removed. Next, the correlation *Q* values were evaluated on the filtered set of localizations and compared to the *Q* values from the found clusters. The value of *R* was chosen to optimize the correspondence between the two sets of Q values, and was found to be *R* = 12 nm for the datasets at hand. The distribution of cluster based localization uncertainties for this value of R turned out to have an average and standard deviation with *σ*_*m*_ = 5.1 nm and Δ*σ* = 1.9 nm, i.e. close to *R* = 2*σ*. This value is somewhat higher than the precision of 3.0 nm found from the localization procedure, probably due to residual drift correction errors.

## Supporting Information

S1 Text Theoretical derivations. Theoretical results for the three-state activation-bleachingmodel for single fluorophores, the number of activations per fluorophore in a mixed Poisson-geometric probability distribution, the effect of labeling stoichiometry, the estimation of the correlation parameter *Q* at high labeling density, and the effect of false negative localizations.
(PDF)

S1 Fig Linearity of switching kinetics. (a) Empirical distributions of the on- and off-times, respectively *t*_*on*_ and *t*_*off*_, of individual fluorophores were obtained for the data of DNA oligomers labeled with single Alexa Fluor 647 dyes on a glass substrate shown in Fig 1. *t*_*on*_ was determined by finding all localizations belonging to the same activation event and determining the time between the first and last localization. (b) The sum of estimated signal photons *n*_*photons*_ from the combined localizations shows a single exponential distribution. (c) *t*_*off*_ was determined as the time interval between subsequent localizations of the same fluorophore as determined by cluster analysis. The distribution of *t*_*on*_ is mono-exponential, the distribution of *t*_*off*_ is reasonably described with a single exponential distribution but possibly also by a bi-exponential distribution, which can possibly be attributed to residual effects of sample contaminations.
(EPS)

S2 Fig Stoichiometry calibration and characterization of neutravidin tetramers labeled with multiple Alexa Fluor 647 labels. (a) Average number of labels per neutravidin tetramer and (b) stoichiometry parameter *μ* as a function of DOL calibrated from cluster brightness statistics and FCS measurements for Phosphate Buffered Saline (PBS) and Oxygen Scavenging Buffer (OSB). Both indicate labeling according to Poisson statistics for DOL values below about 2 and significant quenching effects for higher DOL values. (c) The photon rate during on-events and the photon count per on-event (i.e. localization) do not depend on DOL. This indicates that single emitters are observed in the detected on-events and the brightness and off-switching of these emitters are not affected by nearby emitters on the same tetramer. (d) The time between localizations of the same neutravidin tetramer decreases with DOL indicating that multiple fluorescent labels are observed per tetramer for higher DOL.
(EPS)

S3 Fig Sensitivity of estimation to the calibration of *μ*. The plots show how the estimated number of localizations per site *M* for the sparse neutravidin datasets from Fig 3 would change with the value of *μ* used to fit the correlation parameter *Q*(*t*). Each plot represents the datasets at one DOL value; each solid line represents a single dataset with a red circle indicating the value for *μ* and resulting *M* shown in Fig 3. For all estimates per DOL, ⟨*S*⟩ was kept constant. The estimate of *M* does not vary more than 10% for changes in *μ* smaller than 1, suggesting that the estimation of *M* is quite robust with respect to possible errors in the calibration procedure for *μ*.
(EPS)

S4 Fig Additional examples of quantitative localization microscopy with multiple emitters per labeled site. (a) Image of neutravidin-biotin-phalloidin labeled actin. Note that the switching model parameters *k*_*bl*_ = 5.3 × 10^−3^/s and *M*_∞_ = *k*_*sw*_/*k*_*bl*_ = 3.1 that were found differed a factor of 2 to 3 from the control experiments (Fig 3b). This implies that a counting error on the same order could be made if the control experiments would be used to calibrate these parameters, as is done in e.g. kymograph analysis. Pixel size: 4 nm. (b) Image of a tubulin control samples labeled using secondary antibodies. The secondary to primary DOL was kept low such that most primary antibodies only had a single secondary antibody to enable a comparison to a cluster analysis based ground truth. Analyzing these data assuming a stoichiometry parameter *μ* = DOL = 1.2 and an average number of emitters per site ⟨*S*⟩ = DOL/ [1 − exp(−DOL)] resulted in *k*_*bl*_ = 8.7 × 10^−3^/s and *M*_∞_ = 8.4. Counting estimates on this sample and similar samples for varying numbers of Alexa Fluor 647 fluorophores per antibody gave a RMSE of approximately 10%. Note that for non-sparse secondary antibody labeling there may be a reduced need to calibrate the secondary to primary antibody labeling stoichiometry (see S1 Text section 3, S5 Fig). Pixel size: 4 nm.
(EPS)

S5 Fig Effect of primary and secondary DOL on counting outcome. The plots show the results of an analysis on the dataset of tubulin labeled with a secondary antibody (AB) labeling scheme shown in Fig. 3 of our previous work [18]. The secondary antibodies were conjugated to either Alexa Fluor 647 or Alexa Fluor 750 dyes. The secondary to primary DOL *μ*_1_ and fluorophore to secondary DOL *μ*_2_ were unknown. Thus we investigated how the estimate of the number of localizations per primary antibody would change with different assumptions for *μ*_1_ and *μ*_2_. The measured cumulative number of localizations was fit for the bleaching rate and the correlation parameter *Q* was fit for a range of values for the unknown parameters *μ*_1_ and *μ*_2_, assuming the Poissonian stoichiometry model described in S1 Text section 3. It appears that the exact value of the secondary to primary DOL *μ*_1_ has a very small effect on the final outcome of the counting procedure provided it is larger than about 1.5. The secondary DOL has a bigger impact on the estimate implying that a calibration of the secondary DOL via e.g. absorption spectroscopy or FCS is advisable.
(EPS)

S6 Fig Characterization of sample contaminations. The data of DNA oligomers labeled with single Alexa Fluor 647 dyes in Fig 1 were compared to data from a control sample without DNA oligomers. (a) The single frame brightness and (b) the total photons per “on-event” (i.e. after combining localizations in consecutive frames) in the unlabeled sample are somewhat lower. (c) The off-times *t*_*off*_ between localizations in the labeled sample have the same distribution as the times *t*_*first*_ of the first localizations in the (retained) clusters used for counting. Clusters that were discarded in the counting analysis (i.e. with *t*_*first*_ > 82.5 s or only localizations with less than 5,000 photons) show a substantially different distribution. These clusters are attributed to sample contaminations and have substantially fewer localizations associated with them than localizations that were retained in the cluster analysis for Fig 1 (see (d)).
(EPS)
