# Correction: Quantitative Localization Microscopy: Effects of Photophysics and Labeling Stoichiometry

**DOI:** 10.1371/journal.pone.0139071

**Published:** 2015-09-21

**Authors:** Robert P. J. Nieuwenhuizen, Mark Bates, Anna Szymborska, Keith A. Lidke, Bernd Rieger, Sjoerd Stallinga

In the Results and Methods sections, there are errors in the fourth and tenth equations. The variables M_∞_ should read as (M_∞_- 1). Please view the complete, correct Equation 4 here:

$$Q(t)\;=\;2(M_{\infty }-1)\;(1-\frac{k_{bl}t}{\text{exp}\left(k_{bl}t\right)-1})+\mu M_{\infty }(1-\text{exp}\left(-k_{bl}t\right))$$

Please view the complete, correction Equation 10 here:

$$Q_{model}(t)\;=\;2(M_{\infty }-1)\;(1-\frac{k_{bl}t}{\text{exp}\left(k_{bl}t\right)-1})+\mu M_{\infty }(1-\text{exp}\left(-k_{bl}t\right))$$
