# Supplementary material for: Quantitative Localization Microscopy: Effects of Photophysics and Labeling Stoichiometry
Source: PLoS One. 2015 May 20;10(5):e0127989. doi: 10.1371/journal.pone.0127989 (PMC4439177; doi:10.1371/journal.pone.0127989)
Supplement: S1 Text — Theoretical results for the three-state activation-bleachingmodel for single fluorophores, the number of activations per fluorophore in a mixed Poisson-geometric probability distribution, the effect of labeling stoichiometry, the estimation of the correlation parameter Q at high labeling density, and the effect of false negative localizations. (PDF) [file pone.0127989.s001.pdf]

S1 Text: Effects of photobleaching and labelling stoichiometry on quantitative localization microscopy

March 6, 2015

1 Three-state activation-bleaching model for single fluorophores

We consider switching between three states, the on-state, the off-state, and the bleached state. The switching between the on and off-states is modelled with the Poisson distribution; i.e. in the absence of photobleaching the number of transitions from the off-state to the on-state $P_m^{\rm sw}$ satisfies:

$$P_m^{\rm sw} = \frac{r^m}{m!} \exp\left(-r\right), \tag{1}$$

where $r = k_{\rm sw} t$ with t the time and $k_{\rm sw}$ the switching rate, which is related to the lifetimes of the on and off-states by $1/k_{\rm sw} = \tau_{\rm on} + \tau_{\rm off}$. The bleaching is governed by the geometrical distribution, namely the probability for bleaching at the m-th switching cycle is:

$$P_m^{\rm bl} = b\left(1-b\right)^{m-1}. \tag{2}$$

where $b = k_{\rm sw}/k_{\rm bl}$ is the probability for bleaching during one cycle, with $k_{\rm bl}$ the effective bleaching rate. Intuitively, for small time scales the statistics will be close to the activation dominated Poisson-model, whereas for large times it will be close to the bleaching dominated geometric distribution. For intermediate times t the probability for m activation cycles is the sum of two terms. The first is the product of the probability $P_m^{\rm sw}$ of having m switching cycles and the probability $(1-b)^m$ that the emitter has not bleached in the m switching cycles. The second term is the product of the probability $P_m^{\rm bl}$ of bleaching during the m-th switching cycle and the probability of having at least m switching cycles. In mathematical terms (for $m \geq 1$):

$$P_m = \left(1-b\right)^m \frac{r^m}{m!} \exp\left(-r\right) + b\left(1-b\right)^{m-1} \sum_{n=m}^{\infty} \frac{r^n}{n!} \exp\left(-r\right). \tag{3}$$

For $m = 0$ bleaching does not play a role, so the probability is then given by the Poisson term only:

$$P_0 = \exp\left(-r\right). \tag{4}$$

It may be verified that

$$\sum_{m=0}^{\infty} P_m = 1, \tag{5}$$

so that conservation of probability is satisfied. A rigorous derivation of these expressions for P_m is presented in the next section.

Interestingly, the probability distribution of the number of activation cycles m is equivalent to the distribution of the minimum of two random variables $m_{\rm Poisson}$ and $m_{\rm geometric}$, where $m_{\rm Poisson}$ is Poisson distributed with expectation value r and $m_{\rm geometric}$ follows a geometric distribution with expectation value $1/b$.

The moments of this probability distribution can be calculated from the moment generating

function:

$$
\begin{aligned}
G(a) &= \sum_{m=0}^{\infty} P_m \exp(am) \\
&= \exp(-r) + \sum_{m=1}^{\infty} ((1-b)\exp(a))^m \frac{r^m}{m!} \exp(-r) \\
&\quad + b\exp(a) \sum_{m=1}^{\infty} ((1-b)\exp(a))^{m-1} \sum_{n=m}^{\infty} \frac{r^n}{n!} \exp(-r) \\
&= \exp(r(1-b)\exp(a) - r) \\
&\quad + b\exp(a) \sum_{n=1}^{\infty} \frac{1-((1-b)\exp(a))^n}{1-(1-b)\exp(a)} \frac{r^n}{n!} \exp(-r) \\
&= \frac{b\exp(a) + (1-\exp(a))\exp(r(1-b)\exp(a)-r)}{1-(1-b)\exp(a)}. \qquad (6)
\end{aligned}
$$

The moments follow from the derivatives of this function at $a = 0$:

$$
\begin{aligned}
M_1(t) &= \sum_{m=1}^{\infty} m P_m = \left.\frac{dG(a)}{da}\right|_{a=0} \\
&= \frac{1}{b}\left[1-\exp(-rb)\right], \qquad (7) \\
M_2(t) &= \sum_{m=1}^{\infty} m^2 P_m = \left.\frac{d^2G(a)}{da^2}\right|_{a=0} \\
&= \frac{1}{b}\left[1-\exp(-rb)\right] + \frac{2(1-b)}{b^2}\left[1-\exp(-rb) - rb\exp(-rb)\right], \qquad (8)
\end{aligned}
$$

giving a correlation parameter Q as:

$$
\begin{aligned}
Q(t) &= \frac{M_2(t) - M_1(t)}{M_1(t)} \\
&= \frac{2(1-b)}{b}\left[1 - \frac{rb}{\exp(rb)-1}\right]. \qquad (9)
\end{aligned}
$$

If we define the asymptotic value $M_\infty = \lim_{t\to\infty} M_1(t) = 1/b$ then the results for the average number of activations and for the correlation parameter Q may be written as:

$$
\begin{aligned}
M_1(t) &= M_\infty\left[1-\exp(-k_{\mathrm{bl}}t)\right], \qquad (10) \\
Q(t) &= 2(M_\infty - 1)\left[1 - \frac{k_{\mathrm{bl}}t}{\exp(k_{\mathrm{bl}}t)-1}\right].. \qquad (11)
\end{aligned}
$$

For $k_{\mathrm{bl}}t \ll 1$ we find:

$$
\begin{aligned}
M_1(t) &\approx k_{\mathrm{sw}}t, \qquad (12) \\
M_2(t) - M_1(t) &\approx \left(1 - \frac{1}{M_\infty}\right)(k_{\mathrm{sw}}t)^2, \qquad (13)
\end{aligned}
$$

which is consistent with Poisson statistics provided that $k_{\mathrm{bl}} \ll k_{\mathrm{sw}}$. For $k_{\mathrm{bl}}t \gg 1$ we find constant values:

$$
\begin{aligned}
M_1(t) &\approx M_\infty, \qquad (14) \\
M_2(t) - M_1(t) &\approx 2M_\infty(M_\infty - 1), \qquad (15)
\end{aligned}
$$

consistent with a geometrical distribution with bleaching probability $1/M_\infty = k_{\mathrm{bl}}/k_{\mathrm{sw}}$ per activation cycle. These limiting cases fit with the a priori expectations.

2 Derivation of mixed Poisson-geometric probability distribution

This section presents a derivation of the mixed Poisson-geometric probability distribution in Eq. 3.

The treatment is based on a generalization of the asymmetric Random Telegraph Signal (RTS) model [1], which describes switching between two states. Here a third state is introduced, representing the bleached state, which can in principle be reached from both the on-state and the off-state of the emitter. So, the starting point is the three-state model with state 0 ('off-state'), state 1 ('on state'), and state 2 (bleached state) with four transition rates k_{01} ($0 \rightarrow 1$), k_{10} ($1 \rightarrow 0$), k_{02} ($0 \rightarrow 2$), and k_{12} ($1 \rightarrow 2$). The total decay rate of state 0 is thus $k_0 = k_{01} + k_{02}$, and the decay rate of state 1 is thus $k_1 = k_{10} + k_{12}$. The lifetimes of the on and off states are thus $\tau_{\text{on}} = 1/k_1$ and $\tau_{\text{off}} = 1/k_0$, generally $\tau_{\text{off}} \gg \tau_{\text{on}}$. Suppose the system starts out in state 0 at time $t = 0$. The probability that the emitter remains in state 0 and never is activated is:

$$g_0\left(t\right) = \exp\left(-k_0 t\right), \tag{16}$$

for $t \geq 0$. The probability that the system makes a single jump to state 1 in this time interval is:

$$g_1\left(t\right) = \int_0^t dt'\, g_0\left(t'\right) k_{01} \exp\left(-k_1\left(t-t'\right)\right), \tag{17}$$

the probability that the system makes two jumps and returns to state 0 is:

$$g_2\left(t\right) = \int_0^t dt'\, g_1\left(t'\right) k_{10} \exp\left(-k_0\left(t-t'\right)\right). \tag{18}$$

The probability that the emitter bleaches directly to state 2 is:

$$h_1\left(t\right) = \int_0^t dt'\, g_0\left(t'\right) k_{02}, \tag{19}$$

and the probability it bleaches to state 2 after one transition to state 1 is:

$$h_1\left(t\right) = \int_0^t dt'\, g_1\left(t'\right) k_{12}. \tag{20}$$

Clearly, these probabilities can be calculated by iteration. This is accomplished most easily by application of a Laplace transform:

$$\hat{g}_n\left(s\right) = \int_0^\infty dt\, g_n\left(t\right) \exp\left(-st\right). \tag{21}$$

If so desired, a transition to the Fourier domain can be made by the substitution $s \rightarrow \varepsilon + i\omega$ and taking the limit $\varepsilon \rightarrow 0$ after the inverse (Fourier) transform. We find that for $n = 2m$ even and $n = 2m+1$ odd different relations hold:

$$\hat{g}_{2m}\left(s\right) = \frac{k_{10}}{k_0+s}\hat{g}_{2m-1}\left(s\right), \tag{22}$$

$$\hat{g}_{2m+1}\left(s\right) = \frac{k_{01}}{k_1+s}\hat{g}_{2m}\left(s\right), \tag{23}$$

$$\hat{h}_{2m}\left(s\right) = \frac{k_{02}}{s}\hat{g}_{2m}\left(s\right), \tag{24}$$

$$\hat{h}_{2m+1}\left(s\right) = \frac{k_{12}}{s}\hat{g}_{2m+1}\left(s\right). \tag{25}$$

Starting from $\hat{g}_0(s) = 1/(k_0 + s)$ this leads to the solutions:

$$\hat{g}_{2m}(s) = \frac{\beta^m}{k_0 + s}, \tag{26}$$

$$\hat{g}_{2m+1}(s) = \frac{\beta^{m+1}}{k_{10}}, \tag{27}$$

$$\hat{h}_{2m}(s) = \frac{k_{02}\beta^m}{(k_0 + s)\, s}, \tag{28}$$

$$\hat{h}_{2m+1}(s) = \frac{k_{12}\beta^{m+1}}{k_{10}s}, \tag{29}$$

with:

$$\beta = \frac{k_{01}k_{10}}{(k_0 + s)(k_1 + s)}. \tag{30}$$

The Laplace transform of the probability that the molecule is activated m times during the time interval t now follows as:

$$\begin{aligned}\hat{q}_m(s) &= \hat{g}_{2m-1}(s) + \hat{g}_{2m}(s) + \hat{h}_{2m-1}(s) + \hat{h}_{2m}(s) \\ &= \frac{k_{10}(k_{02} + s) + (k_{12} + s)(k_0 + s)}{k_{10}(k_0 + s)\, s}\beta^m \\ &= \frac{(k_0 + s)(k_1 + s) - k_{01}k_{10}}{k_{10}(k_0 + s)\, s}\beta^m \\ &= \frac{k_1 + s}{k_{10}s}(1 - \beta)\,\beta^m, \end{aligned} \tag{31}$$

and:

$$\hat{q}_0(s) = \hat{g}_0(s) + \hat{h}_0(s) = \frac{k_{02} + s}{(k_0 + s)\, s}. \tag{32}$$

It may be checked that the sum satisfies:

$$\sum_{m=0}^{\infty} \hat{q}_m(s) = \frac{1}{s}, \tag{33}$$

giving that:

$$\sum_{m=0}^{\infty} q_m(t) = \theta(t), \tag{34}$$

implying that conservation of probability applies.

Only the subset of molecules that is activated at least once is accessible to analysis. It follows that we need the renormalized probability distribution:

$$P_m(t) = \frac{q_m(t)}{1 - q_0(\infty)}, \tag{35}$$

for $m \geq 1$ and $P_0(t) = 0$. Here $q_0(t)$ can be found via an inverse Laplace transform:

$$q_0(t) = \frac{k_{02}}{k_0} + \left(1 - \frac{k_{02}}{k_0}\right)\exp(-k_0 t), \tag{36}$$

giving $q_0(\infty) = k_{02}/k_0$ and a normalization factor $1/(1 - q_0(\infty)) = k_0/k_{01}$ leading to a probability distribution (in the Laplace domain):

$$\hat{P}_m(s) = \frac{k_0(k_1 + s)}{k_{01}k_{10}s}(1 - \beta)\,\beta^m, \tag{37}$$

for $m \geq 1$.

An important simplification can be made for times t much larger than $1/k_1 = \tau_{\mathrm{on}}$. In that case we may use the approximation:

$$\hat{P}_m(s) = \frac{k_0 k_1}{k_{01} k_{10} s} (1-\beta) \beta^m, \tag{38}$$

with:

$$\begin{aligned} \beta &= \frac{k_{01} k_{10}}{k_0 k_1 + (k_0 + k_1) s}, & (39) \\ 1 - \beta &= \frac{k_0 k_1 - k_{01} k_{01} + (k_0 + k_1) s}{k_0 k_1 + (k_0 + k_1) s}. & (40) \end{aligned}$$

At this point it is convenient to introduce the two physically relevant rates/time scales, namely the activation and bleaching rates, defined by:

$$\begin{aligned} k_{\mathrm{sw}} &= \frac{k_0 k_1}{k_0 + k_1}, & (41) \\ k_{\mathrm{bl}} &= \frac{k_0 k_1 - k_{01} k_{10}}{k_0 + k_1} = \frac{k_{01} k_{12} + k_{10} k_{02} + k_{02} k_{12}}{k_0 + k_1}. & (42) \end{aligned}$$

The activation time constant is simply $\tau_{\mathrm{sw}} = 1/k_{\mathrm{sw}} = \tau_{\mathrm{on}} + \tau_{\mathrm{off}}$. We also find that $k_{01} k_{10}/k_0 k_1 = 1 - k_{\mathrm{bl}}/k_{\mathrm{sw}}$. Now the probability distribution can be written as:

$$\hat{P}_m(s) = \frac{k_{\mathrm{sw}} (k_{\mathrm{sw}} - k_{\mathrm{bl}})^{m-1} (k_{\mathrm{bl}} + s)}{s (k_{\mathrm{sw}} + s)^{m+1}}, \tag{43}$$

This expression may be rewritten in a form that is more amenable to inverse Laplace transform:

$$\begin{aligned} \hat{P}_m(s) &= \left(1 - \frac{k_{\mathrm{bl}}}{k_{\mathrm{sw}}}\right)^{m-1} \frac{k_{\mathrm{sw}}^m}{(k_{\mathrm{sw}} + s)^{m+1}} + \frac{k_{\mathrm{bl}}}{k_{\mathrm{sw}}} \left(1 - \frac{k_{\mathrm{bl}}}{k_{\mathrm{sw}}}\right)^{m-1} \frac{k_{\mathrm{sw}}^{m+1}}{s (k_{\mathrm{sw}} + s)^{m+1}} \\ &= \left(1 - \frac{k_{\mathrm{bl}}}{k_{\mathrm{sw}}}\right)^{m-1} \frac{k_{\mathrm{sw}}^m}{(k_{\mathrm{sw}} + s)^{m+1}} \\ &\quad + \frac{k_{\mathrm{bl}}}{k_{\mathrm{sw}}} \left(1 - \frac{k_{\mathrm{bl}}}{k_{\mathrm{sw}}}\right)^{m-1} \left[\frac{1}{s} - \sum_{n=1}^{m} \frac{k_{\mathrm{sw}}^n}{(k_{\mathrm{sw}} + s)^{n+1}}\right], \end{aligned} \tag{44}$$

The inverse Laplace transform now gives:

$$\begin{aligned} P_m(t) &= \left(1 - \frac{k_{\mathrm{bl}}}{k_{\mathrm{sw}}}\right)^{m-1} \frac{(k_{\mathrm{sw}} t)^m}{m!} \exp(-k_{\mathrm{sw}} t) \\ &\quad + \frac{k_{\mathrm{bl}}}{k_{\mathrm{sw}}} \left(1 - \frac{k_{\mathrm{bl}}}{k_{\mathrm{sw}}}\right)^{m-1} \left[1 - \sum_{n=0}^{m} \frac{(k_{\mathrm{sw}} t)^n}{n!} \exp(-k_{\mathrm{sw}} t)\right] \\ &= \left(1 - \frac{k_{\mathrm{bl}}}{k_{\mathrm{sw}}}\right)^{m} \frac{(k_{\mathrm{sw}} t)^m}{m!} \exp(-k_{\mathrm{sw}} t) \\ &\quad + \frac{k_{\mathrm{bl}}}{k_{\mathrm{sw}}} \left(1 - \frac{k_{\mathrm{bl}}}{k_{\mathrm{sw}}}\right)^{m-1} \sum_{n=m}^{\infty} \frac{(k_{\mathrm{sw}} t)^n}{n!} \exp(-k_{\mathrm{sw}} t), \end{aligned} \tag{45}$$

in agreement with the results of the previous section.

3 Effect of labelling stoichiometry

Suppose there are K labelling sites with S_i ($i = 1, 2 \ldots K$) fluorescent emitters per site which have M_{ij} activations ($j = 1, 2 \ldots S_i$). The number of activations per site is then:

$$M_i = \sum_{j=1}^{S_i} M_{ij}. \tag{46}$$

Suppose the statistics of the number of emitters per site is independent of the site and has moments $\langle S \rangle$ and $\langle S^2 \rangle$. Suppose furthermore that the statistics of the number of activations of each emitter is independent of emitter and site and gives rise to moments according to the three-state model:

$$\begin{aligned} \langle M_{ij} \rangle &= M_\infty \left[1 - \exp\left(-k_{\text{bl}} t\right)\right], & (47) \\ \langle M_{ij}^2 - M_{ij} \rangle &= 2M_\infty \left(M_\infty - 1\right) \left[1 - \exp\left(-k_{\text{bl}} t\right) - k_{\text{bl}} t \exp\left(-k_{\text{bl}} t\right)\right], & (48) \end{aligned}$$

for all i and j and with $M_\infty = k_{\text{sw}}/k_{\text{bl}}$. The Q-parameter determined from the spatial correlation analysis is given by:

$$Q = \frac{\langle {M_i}^2 - M_i \rangle}{\langle M_i \rangle}, \tag{49}$$

with:

$$\langle M_i \rangle = \langle S \rangle \langle M_{ij} \rangle, \tag{50}$$

and:

$$\langle {M_i}^2 \rangle = \langle S \left(S - 1\right) \rangle \langle M_{ij} \rangle^2 + \langle S \rangle \langle {M_{ij}}^2 \rangle.$$

Combining all results gives:

$$Q = 2\left(M_\infty - 1\right) \left[1 - \frac{k_{\text{bl}} t}{\exp\left(k_{\text{bl}} t\right) - 1}\right] + \mu M_\infty \left[1 - \exp\left(-k_{\text{bl}} t\right)\right], \tag{51}$$

with:

$$\mu = \frac{\langle S^2 \rangle - \langle S \rangle}{\langle S \rangle}, \tag{52}$$

a number characterizing the statistics of the number of emitter per site. The second term on the r.h.s. is new compared to the previous analysis of the statistics per emitter. Clearly, there are now three parameters that determine Q as a function of t, the effective bleaching rate k_{bl}, the asymptotic value of the number of activations per emitter M_∞ and the labelling stoichiometry parameter μ. The expected total number of activations is:

$$\begin{aligned} \langle N \rangle &= \sum_{i=1}^{K} \sum_{j=1}^{S_i} M_{ij} = K \langle S \rangle \langle M_{ij} \rangle \\ &= K \langle S \rangle M_\infty \left[1 - \exp\left(-k_{\text{bl}} t\right)\right]. & (53) \end{aligned}$$

All that is lacking then to determine the number of labelling sites K is a connection between $\langle S \rangle$ and μ. We consider now three examples in which there is a connection between the mean and the variance of the statistical distribution of the number of emitter per site.

The first example refers to having a monomer/dimer on each site with probabilities $P_1 = 1 - \beta$ and $P_2 = \beta$. It follows that then $\langle S \rangle = 1 + \beta$ and $\langle S^2 \rangle = 1 + 3\beta$ so that $\mu =$

$2\beta/\left(1+\beta\right)$. Measurement of μ from Q thus gives a value for $\beta = \mu/\left(2-\mu\right)$ and hence for $\langle S\rangle = 2/\left(2-\mu\right)$. So, the average degree of monomerization/dimerization can potentially be measured in this way, in addition to the total number of labelling sites.

The second example is for a Poisson distributed number of emitters per site. This is a model for primary antibody labelling where multiple fluorophores are attached to the antibody, under the condition that there is no significant fluorescence quenching. The averages are over the subset of sites with at least one emitter. This gives $\langle S\rangle = \eta/\left(1-\exp(-\eta)\right)$ and $\langle S^2 - S\rangle = \eta^2/\left(1-\exp(-\eta)\right)$ with η the Poisson rate, so that $\mu = \eta$. Possibly, the Poisson-rate can thus be measured directly from the fit of the measured Q as a function of t to the model. In case η is large compared to unity then we simply have $\langle S\rangle = \mu = \eta$.

The third example is a model for secondary antibody labelling, where multiple secondaries can bind to a single primary, and where multiple emitters are attached to each secondary, i.e. now $S = \sum_{i=1}^{n} T_i$ with n the number of secondaries and the T_i the number of emitters per secondary. We will analyse the case where the n are Poisson distributed with rate μ_1 and the T_i with rate μ_2. The probability distribution of S is given by:

$$
\begin{aligned}
P\left(S\right) &= \sum_{n=0}^{\infty} P\left(S|n\right) P_1\left(n\right), && (54)\\
P\left(S|n\right) &= \sum_{T_1=0}^{\infty} \ldots \sum_{T_n=0}^{\infty} P_2\left(T_1\right) \ldots P_2\left(T_n\right) \delta\left(S - \sum_{i=1}^{n} T_i\right), && (55)\\
P_1\left(n\right) &= \frac{\mu_1^n \exp\left(-\mu_1\right)}{n!}, && (56)\\
P_2\left(T\right) &= \frac{\mu_2^T \exp\left(-\mu_2\right)}{T!}. && (57)
\end{aligned}
$$

We find that the probability of observing zero fluorophores is:

$$
\begin{aligned}
P\left(0\right) &= \sum_{n=0}^{\infty} P\left(0|n\right) P_1\left(n\right) = \sum_{n=0}^{\infty} \frac{\mu_1^n \exp\left(-\mu_1\right)}{n!} \exp\left(-n\mu_2\right)\\
&= \exp\left(-\mu_1\left(1-\exp\left(-\mu_2\right)\right)\right). && (58)
\end{aligned}
$$

Restricting to the observed cases $S > 0$ implies we have to normalize the probability distribution by a factor $1/\left(1-P\left(0\right)\right)$ and sum only over values $S > 0$. This leads to:

$$
\begin{aligned}
\langle S\rangle &= \frac{1}{1-P\left(0\right)} \langle n\rangle \langle T\rangle, && (59)\\
\left\langle S^2\right\rangle &= \frac{1}{1-P\left(0\right)} \left[\langle n\rangle \langle T^2\rangle + \langle n(n-1)\rangle \langle T\rangle^2\right], && (60)
\end{aligned}
$$

giving:

$$
\mu = \frac{\langle T^2\rangle}{\langle T\rangle} - 1 + \langle T\rangle \left[\frac{\langle n^2\rangle}{\langle n\rangle} - 1\right]. \qquad (61)
$$

Here, the angular brackets indicate averaging over the individual probability distributions for n and for the T_i. For the Poisson-distribution at hand this gives:

$$
\begin{aligned}
\langle S\rangle &= \frac{\mu_1\mu_2}{1-\exp\left(-\mu_1\left(1-\exp\left(-\mu_2\right)\right)\right)}, && (62)\\
\mu &= \mu_2\left(\mu_1+1\right). && (63)
\end{aligned}
$$

Generally prior knowledge on the distribution of secondaries per primary and the distribution of fluorophores per secondary is needed to proceed. It appears that the final counting result is not very sensitive to details of the secondary to primary labelling stoichiometry, i.e. errors in the value of μ_1 are largely compensated by opposite errors in the estimated M_∞, giving a relatively robust estimate for the number of localizations per primary antibody. This can be understood semi-quantitatively as follows. In case there is little bleaching the fitting of M_∞ is dominated to a large extent by the switching regime $k_{\text{bl}} t \ll 1$. Then it holds that:

$$\langle M \rangle \approx \langle S \rangle M_\infty k_{\text{bl}} t, \tag{64}$$

$$Q \approx (\mu + 1) M_\infty k_{\text{bl}} t. \tag{65}$$

So, given the measured correlation parameter Q as a function of time and bleach rate k_{bl}, the product $A = (\mu + 1) M_\infty$ is fixed for all values of μ. It then follows that the estimate for the number of localizations per primary antibody is:

$$\langle M \rangle \approx \frac{\langle S \rangle}{\mu + 1} A k_{\text{bl}} t, \tag{66}$$

so that the stoichiometry only affects the final counting estimate via the ratio:

$$\zeta = \frac{\langle S \rangle}{\mu + 1} = \frac{\langle S \rangle^2}{\langle S^2 \rangle}. \tag{67}$$

It turns out that the functional dependence of ζ on μ_2 hardly changes with μ_1 for the range of values $1 < \mu_1 < 5$, with relative variations on the order of 10%. In fact, the dependence of ζ on μ_2 in the range of values $1 < \mu_2 < 5$ is also rather weak. It should be noted that the current analysis neglects quenching, but in case that can be safely neglected, it does show that the counting analysis is robust against errors in the stoichiometry calibration.

4 Estimation of correlation parameter at high labelling density

In samples with high labelling densities or with tightly clustered labelled molecules, the Q-estimation may be prone to overestimation because it mistakes correlations due to the sample's spatial structure for correlations from repeated localization of the same labelling site. Here we will analyse under which conditions this problem is expected to occur.

The Q-estimation algorithm attempts to fit a model function $H(q)$ to the FRC numerator, which describes the decay in spatial correlations due to localizations of the same labelling site. $H(q)$ depends on the unknown spread of localizations of a single labelling site (i.e. effective localization error) due to localization error, errors in the correction for stage drift and the finite size of the labels, and is parameterized in Eq. 6 as:

$$H(q; \sigma_m, \Delta\sigma) = \frac{1}{\sqrt{1 + 8\pi^2 \Delta\sigma^2 q^2}} \exp\left(-\frac{4\pi^2 \sigma_{\text{m}}^2 q^2}{1 + 8\pi^2 \Delta\sigma^2 q^2} \right), \tag{68}$$

where σ_m is the mean of the effective localization error and $\Delta\sigma$ it's standard deviation.

The FRC's numerator $\nu(q)$ can be expressed as:

$$\nu(q) \propto (NS(q) + Q) H(q), \tag{69}$$

where the term $S(q)$ relating to the sample's spatial structure is equal to:

$$S\left(q\right)=\frac{1}{K^{2}}\int d^{2}q'\left|\hat{\psi}\left(\vec{q}'\right)\right|^{2}\frac{\delta\left(\left|\vec{q}'\right|-q\right)}{2\pi q}, \tag{70}$$

with K the number of labelling sites and $\hat{\psi}\left(\vec{q}\right)$ the Fourier transform of the normalized density of labelling.

The algorithm will have difficulties estimating σ_m and $\Delta\sigma$ when the decay of $S(q)H(q)$ and $QH(q)$ cannot be distinguished very well. This would occur if the decay of $S(q)$ is still larger than Q at the spatial frequency $q = 1/2\pi\sigma_m$ where $QH(q)$ starts to decay appreciably. Thus, we have the criterion:

$$NS(1/2\pi\sigma_m) < Q. \tag{71}$$

If we take as an example structure a line of length L and Gaussian cross-section with full width at half maximum w, then we have [2]:

$$S\left(q\right)\approx\frac{1}{\pi qL}\exp\left(-\frac{\pi^{2}q^{2}w^{2}}{2\log(2)}\right), \tag{72}$$

leading to the criterion for the linear density of labelled sites:

$$\rho_{lin}=\frac{K}{L}<\frac{1}{\sigma_m}\left(\frac{Q}{2M}\exp\left(\frac{w^{2}}{8\log(2)\sigma_m^{2}}\right)\right). \tag{73}$$

If the width of the filaments is on the same order as the width of the localization error distribution and $Q \approx M$, it follows that there should be fewer than one site per $2\sigma_m/e \approx 0.74\sigma_m$. In a more typical scenario $Q \approx 1.5M$ and thus the criterion becomes less than one per $0.5\sigma_m$.

Similarly, for a line with a rectangular cross-section and width w we would have [2]:

$$S\left(q\right)\approx\frac{1}{\pi qL}\left(\frac{\sin\left(\pi qw\right)}{\pi qw}\right)^{2}, \tag{74}$$

and thus we get the criterion:

$$\rho_{lin}<\frac{1}{\sigma_m}\left(\frac{Q}{2M}\right)\left(\frac{\sin\left(w/2\sigma_m\right)}{w/2\sigma_m}\right)^{-2}, \tag{75}$$

or if w equals the full width at half maximum of the localization error distribution that ρ_{lin} should be less than one site per $1.23\sigma_m$ (pessimistic case) or one per $0.82\sigma_m$ (typical case).

Thirdly, if we have a bell-shaped structure that can be described by a Gaussian with standard deviation a, then the number of sites K in the structure should satisfy:

$$K<\frac{Q}{M}\exp(a^{2}/\sigma_m^{2}). \tag{76}$$

Finally, if we have a circular structure with a radius a and K sites, then

$$S(q)=\left(2\frac{J_1(2\pi qa)}{2\pi qa}\right)^{2}, \tag{77}$$

and therefore we obtain the criterion

$$K<\frac{Q}{M}\left(2\frac{J_1(a/\sigma_m)}{a/\sigma_m}\right)^{-2}. \tag{78}$$

For $a \approx \sigma_m$ this gives $K < 1.3$ (pessimistic case) or $K < 1.9$ (typical case). For $a \approx 2\sigma_m$ this becomes $K < 3$ (pessimistic case) or $K < 4.5$ (typical case).

5 Effect of false negative localizations

False negative localizations refer to events where a fluorophore is activated during an acquisition but this fluorescence does not lead to a successful localization by the reconstruction algorithm. This may happen for example if nearby fluorophores are simultaneously active or if the fluorophore is very dim or the event is very short. The consequence of false negative localizations is that the number of localizations per fluorophore does not correspond anymore to the number of activation events. Below we will analyse the consequences assuming that the probabilities for activation events to result in a successful localization P_{loc} are independent and the same for all events.

Firstly, let us consider what happens to the expected number of *localizations* per fluorophore M_{loc}:

$$\langle M_{loc} \rangle = \langle \langle M_{loc} | M \rangle \rangle = \langle M P_{loc} \rangle = P_{loc} \langle M \rangle \, . \tag{79}$$

Here, M denotes the number of *activations* per fluorophore. Similarly, we find that:

$$\left\langle M_{loc}^2 \right\rangle = \left\langle \left\langle M_{loc}^2 | M \right\rangle \right\rangle = P_{loc} \left(1 - P_{loc}\right) \langle M \rangle + P_{loc}^2 \left\langle M^2 \right\rangle , \tag{80}$$

from which it follows that

$$Q \to \frac{\langle M_{loc}^2 - M_{loc} \rangle}{\langle M_{loc} \rangle} = \frac{P_{loc}^2 \langle M^2 - M \rangle}{P_{loc} \langle M \rangle} = P_{loc} Q. \tag{81}$$

Another important consequence of false negative localizations is that a fraction P_0 of all fluorophores is never localized. This fraction is given by:

$$P_0 = \sum_{m=1}^{\infty} b \left(1 - b\right)^{m-1} \left(1 - P_{loc}\right)^m = \frac{b \left(1 - P_{loc}\right)}{1 - \left(1 - b\right)\left(1 - P_{loc}\right)} \tag{82}$$

Finally, it can be shown that the probability distribution for the number of localizations per fluorophore is given by the same expression as in Eq. 3, if the following substitutions are made:

$$r \;\to\; P_{loc} r \tag{83}$$

$$b \;\to\; \frac{b}{1 - (1-b)(1-P_{loc})} = \frac{b}{P_{loc} + b\left(1 - P_{loc}\right)} \tag{84}$$

If $b = 1/M_\infty$ is not too large, then effectively only M_∞ appears to be reduced by a factor P_{loc}. However, because M becomes smaller by the same amount, the accuracy of the estimate for M does not deteriorate much. For example, if $P_{loc} = 80\%$ and $M_\infty = 5$ then the estimate for M would be off by 5%.

References

[1] Fitzhugh R. Statistical Properties of the Asymmetric Random Telegraph Signal, with Applications to Single-Channel Analysis. Mathematical Biosciences. 1983;64:75–89.

[2] Nieuwenhuizen RPJ, Lidke KA, Bates M, Leyton Puig D, Grünwald D, Stallinga S, et al. Measuring Image Resolution in Optical Nanoscopy. Nature Methods. 2013 June;10(6):557–562.
